# Effect of Same-Day Volumetric Modulated Arc Therapy on Resource Utilization in Rapid Access Palliative Radiotherapy Clinics Using a Radiation Oncologist-Initiated Automated Planning Script

**DOI:** 10.7759/cureus.95165

**Published:** 2025-10-22

**Authors:** Emily O'Reilly, Maryam Golshan, Nick Chng, Leigh R Bartha, Leanna Drummond, David Hoegler, Nathan Becker, Benjamin Mou

**Affiliations:** 1 Radiation Oncology, BC Cancer - Vancouver, Vancouver, CAN; 2 Medical Physics, BC Cancer - Kelowna, Kelowna, CAN; 3 Medical Physics, BC Cancer - Prince George, Prince George, CAN; 4 Radiation Therapy, BC Cancer - Kelowna, Kelowna, CAN; 5 Radiation Oncology, BC Cancer - Kelowna, Kelowna, CAN

**Keywords:** palliative care, palliative radiotherapy, planning automation, resource utilization, same-day vmat

## Abstract

Purpose: Rapid access palliative (RAP) radiotherapy (RT) clinics enable patients to access urgent same-day consultation, simulation, and treatment. This study aimed to examine the effect of same-day volumetric modulated arc therapy (VMAT) implementation using the Northern Plan Automation Service Treatment Planning Automation Service (NoPAUSE-TPAS) on patient throughput in RAP-RT clinics at a regional cancer center.

Methods:This retrospective study included all patients seen in RAP-RT clinics between February and August 2024 following the introduction of NoPAUSE-TPAS, compared to a data set from January to July 2019, prior to the introduction of NoPAUSE-TPAS. Baseline characteristics were analyzed using descriptive statistics. Measures of resource utilization and quality pertaining to consultations, computed tomography simulation slots, and treatment delivery were assessed.

Results: RAP-RT clinics saw 202 patients in 2024 and 213 in 2019. In 2019, 195 (91.5%) patients received RT to 249 sites, compared to 189 (93.6%) patients who received RT to 246 sites in 2024. Most patients (n=148 (69.5%) in 2019; n=143 (70.8%) in 2024) received RT to one site. Bone was the most common site treated (n=176 (70.7%) in 2019; n=177 (71.9%) in 2024). The most common fractionation was 8 Gy/1 (n=128 (51.5%) in 2019; n=156 (63.4%) in 2024). Of the bone metastases, 117 (66.5%) were treated with a single fraction in 2019, compared to 144 (81.4%) in 2024. Most patients (n=185 (94.9%) in 2019; n=171 (90.5%) in 2024) started RT the same date as consultation. Within the 2024 cohort, 133 (54.1%) sites were treated with an unplanned technique and 113 (45.9%) with VMAT. Of the sites treated, 192 (78%) were eligible for NoPAUSE-TPAS. Of the eligible sites, 113 (58.9%) received treatment with VMAT, with 96 (84.9%) of these delivered on the same date as consultation. The median time for NoPAUSE-TPAS optimization was 12 minutes.

Conclusions:Same-day VMAT using NoPAUSE-TPAS was implemented in RAP-RT clinics with no scheduling changes impacting patient throughput and similar resource utilization compared to historical data. Utilizing automation technology to improve efficiency can enable same-day VMAT for palliative RT.

## Introduction

Palliative radiotherapy (RT) is an effective strategy in the management of symptoms induced by metastatic disease [1]. Recent trends towards hypofractionated approaches, including single fraction regimens, provide symptomatic benefit, patient convenience, and improved resource utilization within cancer centers [2,3]. Rapid access palliative RT (RAP-RT) clinics were developed several decades ago with the goal of ensuring patients have timely access to palliative RT for symptom management [4]. To maintain same-day delivery of RT, RAP-RT clinics often use unplanned field-based treatment techniques with simple beam arrangements such as a parallel opposed pair (POP) or single direct beam [5,6]. This approach favors expediency and convenience over dose conformality. More conformal RT techniques, such as volumetric modulated arc therapy (VMAT), can significantly reduce radiation dose to surrounding organs at risk (OARs) and reduce treatment-related toxicity compared to a POP beam arrangement and are associated with faster treatment delivery to minimize the time that symptomatic patients are on the treatment couch [7]. However, VMAT is more resource intense due to its need for dosimetry and physics quality assurance (QA), where the hand-off of tasks between team members can limit its use in RAP-RT clinics that require same-day treatment delivery.

More recently, research has focused on strategies to further enhance the workflow within RAP-RT clinics while maintaining the high dosimetric quality of palliative RT plans. This has included a study by Nelissen et al. which endeavored to treat patients without a dedicated computed tomography (CT) simulation scan, by using a combination of pre-treatment diagnostic imaging and online cone beam CT (CBCT) imaging [8]. A study by O'Neil et al. also demonstrated improved efficiency using diagnostic CT scans for treatment planning, with no detriment in plan quality [9]. RAP-RT clinics have also explored developing technology and optimal workflows to deliver same-day palliative RT with VMAT [10].

In British Columbia (BC), Canada, a regional cancer center successfully developed an approach to deliver same-day VMAT as a routine option through their RAP-RT clinics. This workflow utilizes an automated planning script run by radiation oncologists, leveraging technology to improve workflow efficiency by eliminating manual dosimetry performed by planners for simple palliative cases. The primary objective of this study was to examine the effect of same-day VMAT implementation on patient throughput using a radiation oncologist-initiated automated planning script in RAP-RT clinics at a regional cancer center. Secondary objectives included additional resource utilization measures such as unused consultation slots, unused simulation slots, utilization of same-day VMAT, and quality measures such as time between tasks and utilization of single fraction RT for bone metastases. 

## Materials and methods

**Figure 1:**
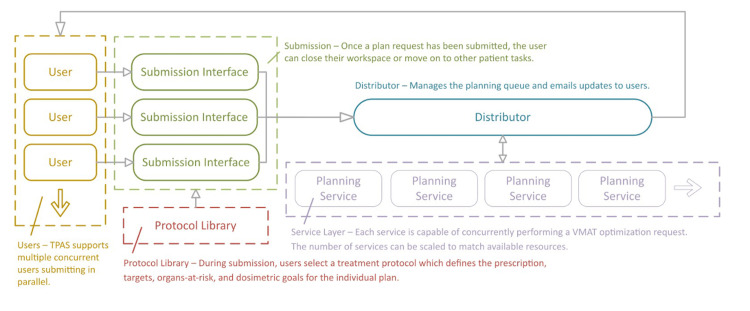
Functional diagram of the Northern Plan Automation Service TPAS. TPAS: Treatment Planning Automation Service; VMAT: volumetric modulated arc therapy

BC Cancer Kelowna is a regional cancer center and the only RT service provider for a large geographic catchment in the southern interior of BC. Since 2017, RAP-RT clinics, comprised of prescheduled consultations, CT simulation scans, and same-day treatment, were scheduled up to four times per week. Each clinic has the capacity to see up to three patients, with each typically receiving treatment to one or two target sites using simple unplanned field-based techniques. In 2024, same-day VMAT was implemented to be routinely available for use in RAP-RT clinics using a radiation oncologist-initiated automated planning script. The Northern Plan Automation Services (NoPAUSE) project included various applications that streamlined treatment planning. One of these was the Treatment Planning Automation Service (TPAS), a platform for single-click VMAT using scripting libraries within the Eclipse v18.0 (Varian, a Siemens Healthineers company, Palo Alto, CA, USA) treatment planning system outlined in Figure 1.

To use NoPAUSE-TPAS, a user submits a planning request through a simple graphic user interface within Eclipse, selecting the specific protocol, prescription dose, target volumes, and OARs. Submitting a request generally takes less than a minute. These requests are sent to a central distributor which manages the center's planning queue and provides email updates to users. The automatic planning process is asynchronous, so users may perform other clinical tasks or submit other requests while waiting for plan optimization. The isocenter and field geometry are determined based on target location and distribution. Optimization is performed on the planning target volume (PTV) and selected OARs to achieve the clinical goals of the protocol, which are reported to the user in an email summary after the process has been completed.

All treatment plans are generated with two arc fields. Depending on the target's location, volume, and contralateral extension, these may consist of two anterior arcs, two partial arcs (left- or right-sided), or two full arcs. The script selects the most appropriate arc geometry based on predefined conditions. For example, anterior arcs are chosen when the target is close to the anterior body surface (i.e., when the source to skin distance exceeds a specified threshold) and when the target does not cross the body midline beyond a set limit (i.e., it is not located too laterally). Arc geometries are evaluated in the following order: anterior, partial (left or right), and finally full arcs. Once a condition is met, the script performs a collision check before confirming that geometry. If a collision is detected, the script advances to the next option. If a collision is also found with the full arc geometry, the arc is cropped by 20° to avoid the collision.

**Figure 2:**
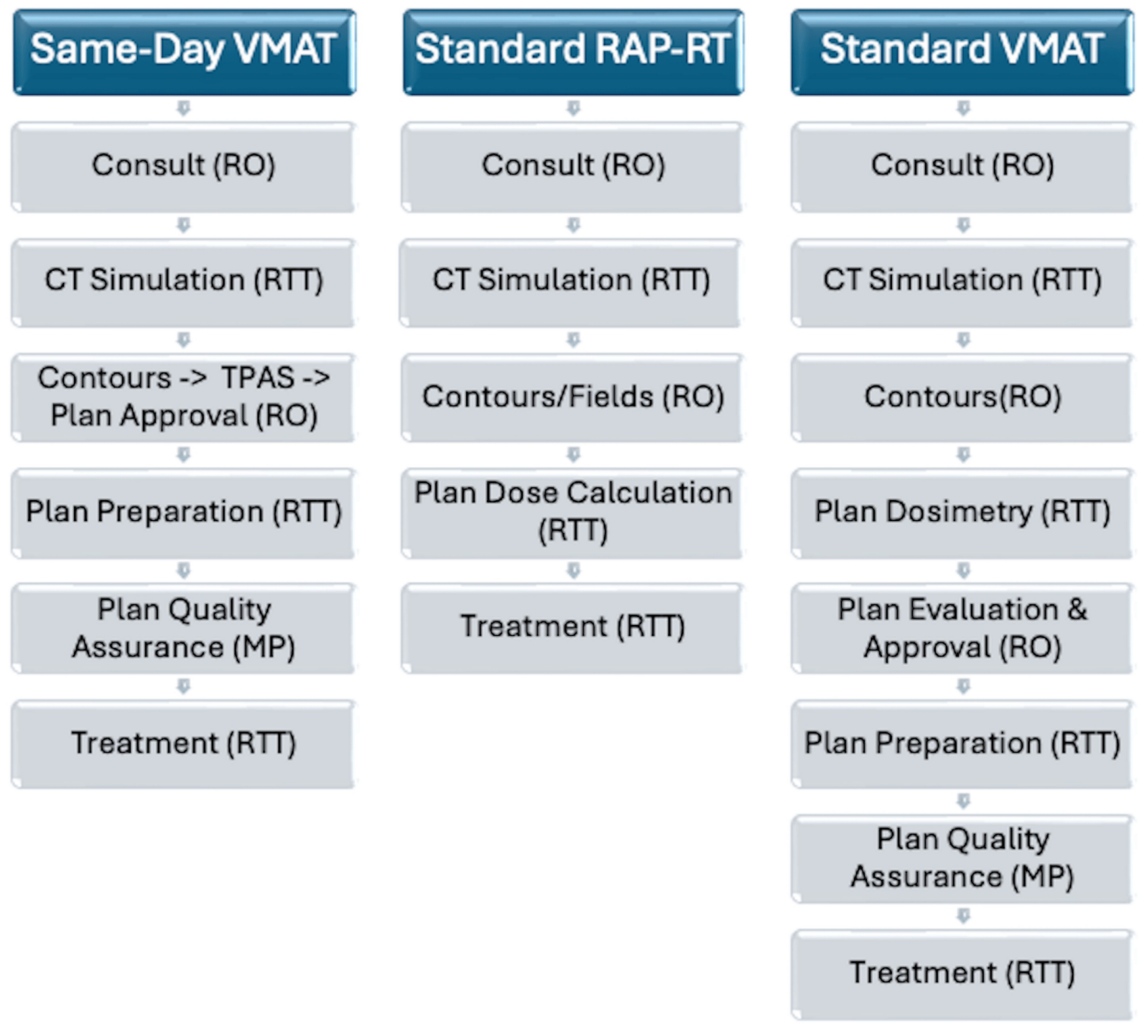
Comparison of clinic workflows. VMAT: volumetric modulated arc therapy; RO: radiation oncologist; CT: computed tomography; TPAS: Treatment Planning Automation Service; RTT: radiation therapist; MP: medical physicist; RAP-RT: rapid access palliative radiotherapy

The clinical goals for all RAP-RT protocols at our center are 95% of the prescription dose to be delivered to greater than 98% of the PTV and PTV maximum dose (Dmax) less than 110% of prescription dose. Up to two OARs may be optionally contoured by the radiation oncologist with a constraint of Dmax less than 110% of the prescription dose. NoPAUSE-TPAS begins with the templated optimization objectives which are iteratively adjusted to achieve those goals using the Eclipse photon optimizer. All plans are analyzed through standard physics QA using Mobius3D (Varian, a Siemens Healthineers company, Palo Alto, CA, USA) and are treated using daily CBCT for image guidance. A comparison of the same-day VMAT workflow with the standard RAP-RT and standard VMAT workflows is outlined in Figure 2.

Patient eligibility for same-day VMAT includes all body sites with expected clinical benefit compared to unplanned field-based treatment. To ensure target and OAR dosimetric goals are achieved and to avoid any delay in treatment, sites that may require more complex VMAT plans are excluded. Additional restrictions to same-day VMAT include simulation and planning tasks that could delay same-day delivery or factors affected by the technical limitations of NoPAUSE-TPAS (Table 1).

**Table 1:** Eligibility criteria for same-day VMAT with NoPAUSE-TPAS. VMAT: volumetric modulated arc therapy; NoPAUSE-TPAS: Northern Plan Automation Service Treatment Planning Automation Service; 4D CT: four-dimensional computed tomography; POP: parallel opposed pair

	Inclusions	Exclusions
Treatment site	Head and neck	Skin (including scalp)
	Spine	Extremities
	Thorax	
	Abdomen	
	Pelvis	
Simulation		4D CT
		Oral or intravenous contrast
		Body width >65 cm at the treatment site
		Pacemaker or implanted cardiac devices within 10 cm along the craniocaudal direction of the treatment site
Dosimetry	Expected clinical benefit compared to unplanned POP beam arrangement	Prosthetics or other hardware in plane of treatment
		Image registration
		Plan sum with previous radiation courses required
Prescription dose	8 Gy in 1 fraction	All other dose fractionations
	20 Gy in 5 fractions	
	30 Gy in 10 fractions	

In the event a same-day VMAT plan was not felt to be clinically acceptable, the radiation oncologist would have the option of treating the patient the same day using unplanned field-based techniques per the standard RAP-RT workflow or rescheduling the patient for treatment five business days later with a manually created VMAT plan per the standard VMAT workflow (Figure 2).

This retrospective study included all patients seen in RAP-RT clinics between February and August 2024 following the introduction of NoPAUSE-TPAS, compared to a data set from January to July 2019, prior to the introduction of NoPAUSE-TPAS. Patients were identified using administrative scheduling codes, specific for RAP-RT clinic consultations, in the Cancer Agency Information System (CAIS) patient record repository. Dates of RAP-RT consultations, CT simulation appointments, and first fraction of RT were extracted from CAIS. Ethics approval was obtained for this study through the University of British Columbia - BC Cancer Research Ethics Board (UBC BC Cancer REB) (approval number: H24-01519).

Baseline patient, tumor, and treatment characteristics were analyzed using descriptive statistics. Patient throughput was calculated by the proportion of patients seen in RAP-RT clinics compared to the total available consultation slots. Utilization rates of same-day VMAT and unplanned field techniques were calculated by the proportion of patients receiving each technique compared to the total patients seen. Utilization of single fraction RT for bone metastases was compared to all patients treated for bone metastases. Additional quality measures included time from consultation to CT simulation scan in number of days, time from consultation to first fraction of RT in number of days, and NoPAUSE-TPAS optimization time in minutes. Lastly, the number of unused consultation slots and CT simulation slots were also evaluated compared to the total consultation and CT simulation slots, respectively. All data analysis was completed using Microsoft Excel (Microsoft Corp., Redmond, WA, USA).

## Results

**Figure 3:**
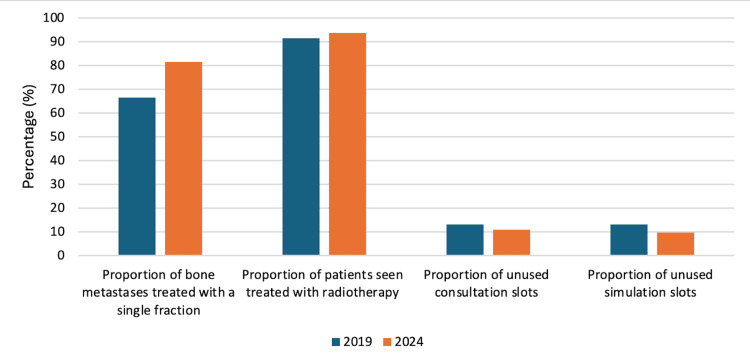
Key findings of resource utilization analysis compared between 2019 and 2024.

Between February and August 2024, 202 patients were seen in RAP-RT clinics compared to 213 patients seen in RAP-RT clinics from January to June 2019. One hundred ninety-five (91.5%) of the patients seen in 2019 received radiation to 249 sites, compared to 189 (93.6%) patients in 2024 who received radiation to 246 sites. The most common histologies were the lung (n=53 (24.9%) in 2019; n=34 (16.8%) in 2024), prostate (n=46 (21.6%) in 2019; n=46 (22.8%) in 2024), and breast (n=38 (17.8%) in 2019; n=37 (18.3%) in 2024). Patients received treatment to one to four treatment sites, with the majority of patients receiving radiation to one site (n=148 (69.5%) in 2019; n=143 (70.8%) in 2024). Bone was the most common site treated, with 176 (70.7%) bone metastases treated in 2019 and 177 (71.9%) bone metastases treated in 2024. The most common dose and fractionation in both cohorts was 8 Gy in one fraction (n=128 (51.4%) in 2019; n=156 (63.4%) in 2024), followed by 20 Gy in five fractions (n=87 (34.9%) in 2019; n=71 (29%) in 2024). One hundred seventeen (66.5%) bone metastases were treated with a single fraction of RT in 2019, compared to 144 (81.4%) in 2024 (Table 2, Figure 3).

**Table 2:** Baseline patient, tumor, and treatment characteristics for patients treated in the RAP-RT clinic in 2019 and 2024. RAP-RT: rapid access palliative radiotherapy; GU: genitourinary

		2019: n (%)	2024: n (%)
Age, median (range)		69 (20-95)	72 (30-92)
Primary histology	Breast	38 (17.8)	37 (18.3)
	Gastrointestinal	19 (8.9)	22 (10.9)
	Gynecological	2 (0.9)	2 (1)
	Head and neck	5 (2.3)	4 (1.9)
	Lung	53 (24.9)	34 (16.8)
	Lymphoma	6 (2.8)	14 (6.9)
	Melanoma	4 (2.8)	2 (1)
	Myeloma	13 (6.1)	21 (10.4)
	Non-prostate GU	17 (7.9)	7 (3.5)
	Prostate	46 (21.6)	46 (22.8)
	Sarcoma		4 (2)
	Other	10 (4.7)	9 (4.5)
Number of sites treated	0	18 (7.8)	13 (6.4)
	1	148 (69.5)	143 (70.8)
	2	41 (19.2)	35 (17.3)
	3	5 (2.3)	11 (5.5)
	4	1 (0.5)	0 (0)
Treatment site	Bone	176 (70.7)	177 (71.9)
	Brain	17 (6.8)	10 (4.1)
	Thorax	36 (14.5)	29 (11.8)
	Abdomen	5 (2)	10 (4.1)
	Pelvis	7 (2.8)	12 (4.8)
	Other	8 (3.2)	8 (3.3)
Dose and fractionation	8 Gy/1 fraction	128 (51.4)	156 (63.4)
	20 Gy/5 fractions	87 (34.9)	71 (29)
	30 Gy/10 fractions	8 (3.2)	5 (2)
	Other	26 (10.4)	14 (5.7)
Proportion of bone metastases treated with single fraction		117 (66.5)	144 (81.4)

There were 245 available radiation oncology consultation and CT simulation slots in 2019 and 227 in 2024. Of these, 32 (13.1%) consultations were unused in 2019 compared to 25 (11%) in 2024. This is compared to 32 (13.1%) unused CT simulation slots in 2019 and 22 (9.7%) in 2024 (Table 3, Figure 3).

**Table 3:** Baseline clinical resource utilization data from the RAP-RT clinic in 2019 and 2024. RAP-RT: rapid access palliative radiotherapy; CT: computed tomography

	2019: n (%)	2024: n (%)
Available radiation oncology consultation and CT simulation slots	245	227
Patients seen	213	202
Patients treated with radiotherapy	195 (91.5)	189 (93.6)
Treatment sites	249	246
Patients new to radiation oncology	80 (37.6)	75 (37.1)
Unused radiation oncology consultation slots	32 (13.1)	25 (11)
Unused CT simulation slots	32 (13.1)	22 (9.7)

The vast majority of patients (n=193 (99%) in 2019; n=186 (98.9%) in 2024) received their CT simulation scan on the same date of their consultation. Of these patients, 185 (94.9%) and 171 (90.5%) also started RT on the same date of their consultation in 2019 and 2024, respectively. The most common reasons for a patient to not receive RT within RAP-RT clinics included lack of symptoms at the time of consultation or requiring dosimetry for planning purposes. The most common reasons for delays were due to the timing of systemic therapy, requiring dosimetry for planning, or pursuing a more aggressive RT approach with a higher prescription dose than available using NoPAUSE-TPAS (Table 4). 

**Table 4:** Timing of CT simulation and first fraction of radiotherapy relative to radiation oncology consultation. CT: computed tomography

		2019: n (%)	2024: n (%)
Days from consultation to CT simulation	0	193 (99)	186 (98.9)
	1		2 (1.1)
	7	1 (0.5)	
	23	1 (0.5)	
Days from consultation to first fraction	0	185 (94.9)	171 (90.5)
	1	3 (1.6)	7 (3.7)
	3		2 (1.1)
	4-31	6 (3.2)	9 (4.8)
Reasons for no radiotherapy	Asymptomatic	1 (5.6)	6 (46.2)
	Patient preference	3 (16.7)	1 (7.7)
	Normal tissue tolerance or dosimetry required	6 (33.3)	2 (15.4)
	Poor performance status	3 (16.7)	1 (7.7)
	Alternative treatment modality	2 (11.1)	2 (15.4)
	Biopsy required	1 (5.6)	1 (7.7)
	Pain crisis	1 (5.6)	
	Location of pain unrelated to cancer	1 (5.6)	
Reasons for delay in radiotherapy	Systemic therapy timing	2 (22.2)	1 (5.6)
	Pain crisis	1 (11.1)	
	Biopsy required	1 (11.1)	
	Tumor board review	1 (11.1)	1 (5.6)
	Hospital admission		1 (5.6)
	Patient preference		1 (5.6)
	Dosimetry required		9 (50)
	Unknown	4 (44.4)	5 (27.6)

Within the 2024 cohort, 133 (54.1%) sites were treated with an unplanned technique and 113 (45.9%) sites were treated with VMAT. Of the total sites, 192 (78%) were eligible for NoPAUSE-TPAS, with 113 (58.9%) of the eligible sites receiving treatment with VMAT. Ninety-six (84.9%) VMAT treatments were delivered on the same day as consultation. The median time for radiation oncologist-initiated VMAT optimization time was 12 minutes (interquartile range, 9-15 minutes). Four cases were reported as optimization failures by the system. All four of these were re-optimized on a second attempt, with a median optimization time of 12 minutes (interquartile range, 11-12.5 minutes) (Table 5).

**Table 5:** Radiotherapy planning and NoPAUSE-TPAS utilization data for patients treated in the RAP-RT clinic in 2024. NoPAUSE-TPAS: Northern Plan Automation Service Treatment Planning Automation Service; RAP-RT: rapid access palliative radiotherapy; VMAT: volumetric modulated arc therapy; IQR: interquartile range

	n (%)
TPAS eligible sites	192 (78)
Unplanned-radiotherapy technique	133 (54.1)
VMAT-radiotherapy technique	113 (45.9)
Proportion of VMAT plans in NoPAUSE-TPAS eligible sites	113 (58.9)
Proportion of VMAT plans delivered on the same day as consultation	96 (84.9)
Median NoPAUSE-TPAS optimization times in minutes (IQR)	12 (9-15)
Proportion of NoPAUSE-TPAS failures	4 (3.5)

## Discussion

Same-day VMAT using a radiation oncologist-initiated automated planning script in a regional cancer center was feasible with no adverse impacts on patient throughput or resource utilization. The maintenance of same-day RT delivery was a driving factor behind the clinical workflow and eligibility criteria for same-day VMAT to ensure patients received VMAT in the same timeframe as an unplanned field-based technique. Specific to the catchment of Kelowna, BC, the majority of the patient population lives more than a two-hour drive one way to the cancer center, further stressing the importance of maintaining prompt access to palliative RT to minimize financial and logistical barriers for rural patients [11]. 

With the radiation oncologist as the primary NoPAUSE-TPAS user, rather than a dosimetrist or physicist, same-day VMAT minimized delays stemming from the transfer of tasks between team members which could compromise same-day treatment delivery (Figure 2). This allowed same-day VMAT to maintain a similar workflow as used in standard RAP-RT clinics.

In this resource utilization study, 113 (58.9%) of the sites eligible for NoPAUSE-TPAS in the 2024 cohort were treated with same-day VMAT. As this was a new process for many radiation oncologists, there may have been an inherent learning curve associated with using the automated script, which may have contributed to lower utilization rates. With increased familiarity with the automated script, it is anticipated that this learning curve will lessen, leading to a subsequent rise in utilization.

Of the cases treated with same-day VMAT, the median optimization time was 12 minutes (interquartile range, 9-15 minutes). Four plans in the 2024 cohort failed NoPAUSE-TPAS optimization. On further review, two failures were due to user modifications made during script execution, one was due to incorrect protocol selection, and another resulted from incorrect data input. All four of these minor errors were easily corrected, and the cases were successfully replanned using NoPAUSE-TPAS on a second attempt without complications with a median optimization time of 12 minutes (interquartile range, 11-12.5 minutes). 

The feasibility of same-day VMAT in RAP-RT clinics has also been studied by Dennis et al. using an anthropomorphic phantom and anonymized planning CT image sets from five previously treated patients with vertebral metastases [10]. Using a stopwatch to measure the elapsed time for each task including contouring, planning, physics QA, and treatment delivery, they found that these tasks could be completed within two hours by experienced staff and concluded same-day VMAT was feasible in their existing RAP-RT clinic model which typically moved patients from CT simulation to treatment in 4-6 hours [10]. In contrast to the current study, planning was performed by an experienced dosimetrist, and planning times ranged from nine to 31 minutes with longer planning times for targets involving the lumbar spine compared to the thoracic spine [10]. The use of an automated planning script may result in more consistent planning times. Furthermore, by having the radiation oncologist run the automated planning script immediately after contouring, additional downtime waiting for tasks to be passed off or picked up by team members can be minimized.

Numerous automated VMAT planning techniques for a wide range of body sites have already been described, and continued advancements will have a growing impact on RT clinics in the future [12-18]. These technological advances can lead to more efficient workflows where planners can spend more time and effort on complex palliative cases, such as stereotactic body RT for bone metastases [19,20]. The use of scripts may also assist in generating more consistent plans over a shorter period of time. For most palliative treatments, the incorporation of automated planning can generate a rapid simple VMAT plan by radiation oncologists in several minutes. This allows VMAT to be reliably and routinely delivered on the same day in RAP-RT clinics without excessive resource requirements. In the setting of finite resources, RT centers must balance improvements in turnaround times with safety and appropriate resource utilization. This may involve triaging palliative cases into different levels of complexity; however, the ability to offer same-day VMAT in RAP-RT clinics can significantly improve the therapeutic ratio for patients to receive timely palliative RT with fewer treatment-related side effects. An ongoing randomized phase III trial is investigating the extent of symptom control with VMAT compared to simple unplanned techniques in patients receiving palliative RT and whether the additional resources associated with VMAT would be justified to improve patient toxicity outcomes [21]. As we await the definitive results of this trial, RT technology continues to advance, and the growing use of automated planning using scripts can significantly improve efficiency even in low-resource environments.

Limitations of this study include the scope of resource utilization being primarily limited to resources pertaining to consultations, CT simulation slots, and treatment delivery. Other resources not accounted for include physicist QA time which was non-existent for unplanned field-based treatments, but was comparatively lower than for typical complex VMAT plans. Additionally, the data from time stamps was limited to VMAT optimization times. Although the time stamps provide an overview of the NoPAUSE-TPAS optimization times for each case, they do not capture information related to non-planning tasks in the clinical environment which may impact the ability to deliver same-day palliative RT. Furthermore, the cohort examined in this study was from the first six months following the implementation of NoPAUSE-TPAS. It is therefore possible that the utilization rates of TPAS for eligible cases may have initially been lower secondary to an inherent learning curve associated with the implementation of the automated script. Finally, this study was conducted in a jurisdiction with a publicly funded single-payer healthcare system where physicians and clinic staff are salaried. The feasibility of creating a same-day palliative VMAT program may not be generalizable to institutions with different reimbursement models affected by RT technique. However, this study provides a platform for further investigation and potential further implementation of same-day VMAT in other RAP-RT clinical settings.

## Conclusions

Same-day VMAT using an automated planning script was feasible and implemented for routine use in RAP-RT clinics at a regional cancer center with a large rural population. Implementation of same-day VMAT did not lead to any scheduling changes which would have adversely impacted patient throughput and had similar resource utilization compared to historical data. Utilizing automation technology to improve efficiency with a modest change in resources can permit same-day VMAT, which can improve the therapeutic ratio for patients receiving palliative RT. Utilization of same-day VMAT may also have important implications for resource-limited areas and to help minimize travel time for patients residing in rural or remote communities. Continued efforts to streamline workflows to deliver prompt high-quality palliative RT are encouraged.
